# 4-[(*E*)-3-Meth­oxy-5-nitro-4-(4-nitro­benz­yloxy)benzyl­idene­amino]-1,5-dimethyl-2-phenyl-1*H*-pyrazol-3(2*H*)-one

**DOI:** 10.1107/S1600536810039152

**Published:** 2010-10-09

**Authors:** Lei Liao

**Affiliations:** a School of Manufacturing Science and Engineering, Southwest University of Science and Technology, Mianyang City, Sichuan Province 621010, People’s Republic of China

## Abstract

In the title compound, C_26_H_23_N_5_O_7_, the central benzene ring makes dihedral angles of 35.08 (6), 48.75 (7) and 69.55 (8)° with the pyrazolone ring, the nitro­benzene ring and the terminal phenyl ring, respectively. An intra­molecular C—H⋯O inter­action generates an *S*(6) ring. The packing is stabilized by weak nonclassical inter­molecular C—H⋯O=C hydrogen bonds that link adjacent mol­ecules into chains.

## Related literature

For general background to related compounds, see: Chen & Yu (2006 ▶); Li *et al.* (2005 ▶); Santos *et al.* (2001 ▶); Zhang *et al.* (2006 ▶). For reference bond lengths, see: Allen *et al.* (1987 ▶).
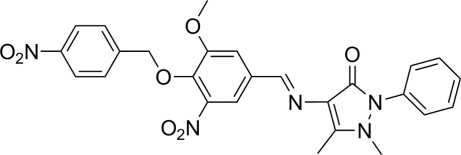


## Experimental

### 

#### Crystal data

C_26_H_23_N_5_O_7_
*M* *_r_* = 517.49
Monoclinic, 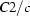

*a* = 25.671 (11) Å
*b* = 11.933 (5) Å
*c* = 16.617 (7) Å
β = 103.972 (7)°
*V* = 4940 (4) Å^3^
*Z* = 8
Mo *K*α radiation
μ = 0.10 mm^−1^
*T* = 294 K
0.20 × 0.18 × 0.10 mm

#### Data collection

Bruker SMART APEX CCD diffractometer
Absorption correction: multi-scan (*SADABS*; Sheldrick, 1996 ▶) *T*_min_ = 0.938, *T*_max_ = 0.989
12593 measured reflections
4363 independent reflections
2652 reflections with *I* > 2σ(*I*)
*R*_int_ = 0.039

#### Refinement

*R*[*F* ^2^ > 2σ(*F* ^2^)] = 0.048
*wR*(*F* ^2^) = 0.139
*S* = 1.02
4363 reflections
346 parameters
H-atom parameters constrained
Δρ_max_ = 0.39 e Å^−3^
Δρ_min_ = −0.21 e Å^−3^

### 

Data collection: *SMART* (Bruker, 1999 ▶); cell refinement: *SAINT* (Bruker, 1999 ▶); data reduction: *SAINT*; program(s) used to solve structure: *SHELXS97* (Sheldrick, 2008 ▶); program(s) used to refine structure: *SHELXL97* (Sheldrick, 2008 ▶); molecular graphics: *SHELXTL* (Sheldrick, 2008 ▶); software used to prepare material for publication: *SHELXTL*.

## Supplementary Material

Crystal structure: contains datablocks I, global. DOI: 10.1107/S1600536810039152/hb5658sup1.cif

Structure factors: contains datablocks I. DOI: 10.1107/S1600536810039152/hb5658Isup2.hkl

Additional supplementary materials:  crystallographic information; 3D view; checkCIF report

## Figures and Tables

**Table 1:** Hydrogen-bond geometry (Å, °)

*D*—H⋯*A*	*D*—H	H⋯*A*	*D*⋯*A*	*D*—H⋯*A*
C15—H15⋯O7	0.93	2.35	2.997 (3)	127
C4—H4⋯O7^i^	0.93	2.56	3.447 (3)	159

Symmetry code: (i) 
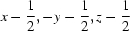
.

**Fig. 1.**
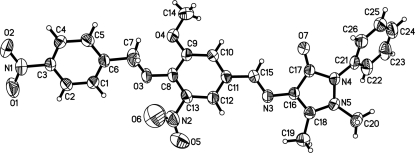
The structure of (I), with displacement ellipsoids for non-H atoms drawn at the 50% probability level.

**Fig. 2.**
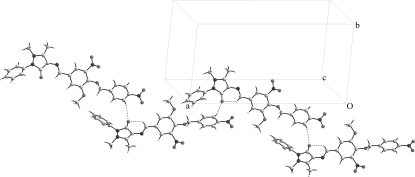
A packing diagram for (I), with hydrogen bonds drawn as dashed lines.

## supplementary crystallographic information

### Comment

Schiff bases have extensively been studied because of their significant biological activity such as protein and enzyme mimics (Santos *et al.*, 2001). Consequently, many Schiff base derivatives have been synthesized and employed to develop protein and enzyme mimics such as models to mimic hydrolase in the hydrolysis of *p*-nitrophenyl picolinate (Li *et al.*, 2005).

Among the large number of compounds, 4-amino-1,5-dimethyl-2-phenylpyrazol-3-one forms a variety of Schiff bases with aldehydes, and the synthesis and crystal structures of some of them, such as (*E*)-4-(4-(4-chlorobenzyloxy)-3-ethoxybenzylideneamino) -1,5-dimethyl-2-phenyl-1,2-dihydropyrazol-3-one (Zhang *et al.*, 2006) and (*E*)-2-(2-((1,5-dimethyl-3-oxo-2-phenyl-2,3-dihydro-1*H*-pyrazol-4-ylimino) methyl)phenoxy)ethyl 4-methylbenzenesulfonate(Chen & Yu, 2006) have been reported.

Structural information is useful when investigating the coordination properties of Schiff bases functioning as ligands. We report here the synthesis and molecular structure of the title Schiff base compound, (I), (Fig. 1)

In the title molecule (Fig. 1), bond lengths and angles are within normal ranges (Allen *et al.*, 1987). The pyrazolone ring (C16—C18/N3—N5/O7) is nearly planar, with an r.m.s. deviation for fitted atoms of 0.0379 Å. It makes a dihedral angle of 44.37 (9)° with the attached phenyl ring (C21—C26). The central benzene ring (C8—C13/C15/O3/O4) is almost planar, with an r.m.s. deviation for fitted atoms of 0.0342 Å. This group makes dihedral angles of 35.08 (6)°, 48.75 (7)° and 69.55 (8)°, respectively, with the the pyrazolone ring (C16—C18/N3—N5/O7), the nitrobenzene ring (C1—C6) and the terminal phenyl ring (C21—C26).

An intramolecular C15—H15···O7═C17 hydrogen bond is found in (I) (Table 1), which helps to stabilize the conformation of the molecule. Packing is stabilized by weak, non-classical intermolecular C4—H4···O7═C117 hydrogen bonds that link adjacent molecules into one-dimensional chains (Table 1, Fig. 2).

### Experimental

An anhydrous ethanol solution (100 ml) of 3-methoxy-5-nitro-4-(4-nitrobenzyloxy)benzaldehyde (3.32 g, 10 mmol) was added to an anhydrous ethanol solution (50 ml) of 4-amino-1,5-dimethyl-2-phenylpyrazol-3-one (2.03 g, 10 mmol) and the mixture stirred at 350 K for 3 h under N_2_, giving a yellow precipitate. The product was isolated, recrystallized from acetonitrile, and then dried in a vacuum to give pure compound (I) in 79% yield. Yellow blocks of (I) were obtained by slow evaporation of an acetonitrile solution.

### Refinement

The H atoms were included in calculated positions and refined using a riding model approximation. Constrained C—H bond lengths and isotropic U parameters: 0.93 Å and *U*_iso_(H) = 1.2*U*_eq_(C) for C*sp*^2^—H; 0.97 Å and *U*_iso_(H) = 1.2*U*_eq_(C) for methylene C—H; 0.96 Å and *U*_iso_(H) = 1.5*U*_eq_(C) for methyl C—H.

### Crystal data

**Table:**

C_26_H_23_N_5_O_7_	*F*(000) = 2160
*M**_r_* = 517.49	*D*_x_ = 1.392 Mg m^−^^3^
Monoclinic, *C*2/*c*	Mo *K*α radiation, λ = 0.71073 Å
Hall symbol: -C 2yc	Cell parameters from 3086 reflections
*a* = 25.671 (11) Å	θ = 2.5–23.1°
*b* = 11.933 (5) Å	µ = 0.10 mm^−^^1^
*c* = 16.617 (7) Å	*T* = 294 K
β = 103.972 (7)°	Block, yellow
*V* = 4940 (4) Å^3^	0.20 × 0.18 × 0.10 mm
*Z* = 8	

### Data collection

**Table:**

Bruker SMART APEX CCD diffractometer	4363 independent reflections
Radiation source: fine-focus sealed tube	2652 reflections with *I* > 2σ(*I*)
graphite	*R*_int_ = 0.039
φ and ω scans	θ_max_ = 25.0°, θ_min_ = 1.6°
Absorption correction: multi-scan (*SADABS*; Sheldrick, 1996)	*h* = −30→30
*T*_min_ = 0.938, *T*_max_ = 0.989	*k* = −10→14
12593 measured reflections	*l* = −18→19

### Refinement

**Table:**

Refinement on *F*^2^	Primary atom site location: structure-invariant direct methods
Least-squares matrix: full	Secondary atom site location: difference Fourier map
*R*[*F*^2^ > 2σ(*F*^2^)] = 0.048	Hydrogen site location: inferred from neighbouring sites
*wR*(*F*^2^) = 0.139	H-atom parameters constrained
*S* = 1.02	*w* = 1/[σ^2^(*F*_o_^2^) + (0.0693*P*)^2^ + 1.2662*P*] where *P* = (*F*_o_^2^ + 2*F*_c_^2^)/3
4363 reflections	(Δ/σ)_max_ = 0.001
346 parameters	Δρ_max_ = 0.39 e Å^−^^3^
0 restraints	Δρ_min_ = −0.21 e Å^−^^3^

### Special details

**Table:**

Geometry. All e.s.d.'s (except the e.s.d. in the dihedral angle between two l.s. planes) are estimated using the full covariance matrix. The cell e.s.d.'s are taken into account individually in the estimation of e.s.d.'s in distances, angles and torsion angles; correlations between e.s.d.'s in cell parameters are only used when they are defined by crystal symmetry. An approximate (isotropic) treatment of cell e.s.d.'s is used for estimating e.s.d.'s involving l.s. planes.
Refinement. Refinement of *F*^2^ against ALL reflections. The weighted *R*-factor *wR* and goodness of fit *S* are based on *F*^2^, conventional *R*-factors *R* are based on *F*, with *F* set to zero for negative *F*^2^. The threshold expression of *F*^2^ > 2σ(*F*^2^) is used only for calculating *R*-factors(gt) *etc*. and is not relevant to the choice of reflections for refinement. *R*-factors based on *F*^2^ are statistically about twice as large as those based on *F*, and *R*- factors based on ALL data will be even larger.

### Fractional atomic coordinates and isotropic or equivalent isotropic displacement parameters (Å^2^)

**Table:**

	*x*	*y*	*z*	*U*_iso_*/*U*_eq_	
N1	0.17786 (9)	−0.1619 (2)	−0.12948 (15)	0.0577 (6)	
N2	0.45460 (10)	0.1575 (2)	0.1025 (2)	0.0746 (8)	
N3	0.65269 (7)	0.10798 (16)	0.27094 (12)	0.0441 (5)	
N4	0.79727 (7)	0.09102 (16)	0.33957 (12)	0.0442 (5)	
N5	0.78530 (8)	0.18315 (16)	0.38510 (13)	0.0456 (5)	
O1	0.14745 (8)	−0.0959 (2)	−0.10853 (15)	0.0924 (8)	
O2	0.16274 (8)	−0.23197 (19)	−0.18340 (15)	0.0838 (7)	
O3	0.41585 (6)	−0.06411 (14)	0.08900 (11)	0.0522 (5)	
O4	0.48334 (7)	−0.23900 (15)	0.14053 (13)	0.0675 (6)	
O5	0.45720 (10)	0.2406 (2)	0.1478 (2)	0.1103 (9)	
O6	0.42595 (15)	0.1535 (3)	0.0360 (2)	0.1733 (17)	
O7	0.74844 (6)	−0.04709 (15)	0.25573 (11)	0.0559 (5)	
C1	0.30688 (10)	−0.06890 (19)	0.00891 (16)	0.0471 (6)	
H1	0.3190	−0.0199	0.0530	0.057*	
C2	0.25251 (10)	−0.0791 (2)	−0.02563 (16)	0.0471 (6)	
H2	0.2279	−0.0369	−0.0056	0.057*	
C3	0.23550 (9)	−0.15309 (19)	−0.09037 (15)	0.0402 (6)	
C4	0.27074 (10)	−0.2174 (2)	−0.12056 (16)	0.0479 (6)	
H4	0.2584	−0.2679	−0.1636	0.057*	
C5	0.32471 (10)	−0.2054 (2)	−0.08570 (16)	0.0524 (7)	
H5	0.3491	−0.2483	−0.1058	0.063*	
C6	0.34367 (9)	−0.1309 (2)	−0.02133 (15)	0.0442 (6)	
C7	0.40340 (11)	−0.1185 (3)	0.01048 (19)	0.0792 (10)	
H7A	0.4201	−0.1919	0.0156	0.095*	
H7B	0.4176	−0.0752	−0.0287	0.095*	
C8	0.46952 (9)	−0.0463 (2)	0.12145 (15)	0.0445 (6)	
C9	0.50518 (9)	−0.1341 (2)	0.14976 (16)	0.0454 (6)	
C10	0.55822 (9)	−0.1122 (2)	0.18748 (16)	0.0462 (6)	
H10	0.5818	−0.1716	0.2042	0.055*	
C11	0.57716 (9)	−0.0028 (2)	0.20097 (15)	0.0411 (6)	
C12	0.54226 (9)	0.0855 (2)	0.17466 (15)	0.0455 (6)	
H12	0.5539	0.1593	0.1839	0.055*	
C13	0.48995 (9)	0.0621 (2)	0.13456 (16)	0.0459 (6)	
C14	0.51973 (13)	−0.3321 (2)	0.1583 (2)	0.0860 (11)	
H14A	0.5425	−0.3321	0.1202	0.129*	
H14B	0.4997	−0.4007	0.1528	0.129*	
H14C	0.5414	−0.3256	0.2140	0.129*	
C15	0.63347 (9)	0.0136 (2)	0.24335 (16)	0.0452 (6)	
H15	0.6563	−0.0480	0.2505	0.054*	
C16	0.70778 (9)	0.11570 (18)	0.30688 (14)	0.0391 (6)	
C17	0.74983 (9)	0.0415 (2)	0.29486 (15)	0.0418 (6)	
C18	0.73083 (9)	0.20013 (19)	0.35782 (15)	0.0414 (6)	
C19	0.70450 (11)	0.3001 (2)	0.38397 (17)	0.0575 (7)	
H19A	0.7146	0.3658	0.3580	0.086*	
H19B	0.7157	0.3080	0.4431	0.086*	
H19C	0.6662	0.2910	0.3677	0.086*	
C20	0.82277 (11)	0.2781 (2)	0.39304 (18)	0.0595 (7)	
H20A	0.8205	0.3102	0.3394	0.089*	
H20B	0.8587	0.2522	0.4160	0.089*	
H20C	0.8135	0.3337	0.4290	0.089*	
C21	0.84699 (9)	0.03300 (19)	0.36710 (16)	0.0447 (6)	
C22	0.87379 (10)	0.0311 (2)	0.45004 (18)	0.0596 (7)	
H22	0.8601	0.0695	0.4891	0.072*	
C23	0.92118 (12)	−0.0284 (3)	0.4743 (2)	0.0748 (10)	
H23	0.9394	−0.0296	0.5299	0.090*	
C24	0.94167 (12)	−0.0855 (3)	0.4174 (3)	0.0758 (10)	
H24	0.9734	−0.1258	0.4346	0.091*	
C25	0.91524 (11)	−0.0832 (2)	0.3348 (2)	0.0677 (9)	
H25	0.9292	−0.1219	0.2962	0.081*	
C26	0.86791 (10)	−0.0234 (2)	0.30924 (19)	0.0549 (7)	
H26	0.8502	−0.0212	0.2534	0.066*	

### Atomic displacement parameters (Å^2^)

**Table:**

	*U*^11^	*U*^22^	*U*^33^	*U*^12^	*U*^13^	*U*^23^
N1	0.0418 (14)	0.0746 (16)	0.0518 (15)	−0.0035 (12)	0.0019 (12)	0.0040 (13)
N2	0.0522 (16)	0.0634 (18)	0.095 (2)	0.0082 (13)	−0.0074 (15)	0.0167 (16)
N3	0.0350 (12)	0.0457 (12)	0.0496 (13)	−0.0031 (9)	0.0065 (10)	0.0016 (10)
N4	0.0339 (12)	0.0459 (12)	0.0482 (13)	−0.0017 (9)	0.0009 (9)	−0.0022 (10)
N5	0.0396 (12)	0.0440 (12)	0.0499 (13)	−0.0071 (9)	0.0045 (10)	−0.0059 (10)
O1	0.0398 (12)	0.141 (2)	0.0917 (18)	0.0139 (13)	0.0061 (11)	−0.0275 (15)
O2	0.0578 (14)	0.0940 (16)	0.0865 (17)	−0.0192 (11)	−0.0082 (12)	−0.0233 (13)
O3	0.0289 (9)	0.0750 (12)	0.0497 (11)	0.0002 (8)	0.0037 (8)	−0.0143 (9)
O4	0.0459 (11)	0.0504 (11)	0.0977 (16)	−0.0049 (9)	0.0007 (10)	−0.0121 (10)
O5	0.0948 (19)	0.0689 (16)	0.159 (3)	0.0270 (14)	0.0149 (17)	0.0107 (17)
O6	0.175 (3)	0.122 (2)	0.155 (3)	0.037 (2)	−0.093 (3)	0.025 (2)
O7	0.0407 (10)	0.0532 (11)	0.0691 (13)	−0.0016 (8)	0.0043 (9)	−0.0156 (10)
C1	0.0434 (16)	0.0509 (15)	0.0441 (16)	−0.0005 (12)	0.0050 (12)	−0.0112 (12)
C2	0.0387 (15)	0.0512 (15)	0.0504 (16)	0.0070 (11)	0.0089 (12)	−0.0030 (13)
C3	0.0353 (14)	0.0419 (14)	0.0409 (15)	−0.0028 (11)	0.0043 (11)	0.0061 (11)
C4	0.0480 (16)	0.0460 (15)	0.0449 (16)	−0.0012 (12)	0.0020 (12)	−0.0068 (12)
C5	0.0468 (17)	0.0593 (17)	0.0493 (17)	0.0116 (12)	0.0082 (13)	−0.0090 (13)
C6	0.0351 (14)	0.0553 (15)	0.0395 (15)	0.0038 (11)	0.0037 (11)	−0.0042 (12)
C7	0.0435 (18)	0.134 (3)	0.057 (2)	0.0033 (17)	0.0059 (15)	−0.0360 (19)
C8	0.0284 (13)	0.0637 (17)	0.0404 (15)	−0.0015 (12)	0.0061 (11)	−0.0036 (12)
C9	0.0333 (14)	0.0495 (15)	0.0525 (17)	−0.0038 (12)	0.0087 (12)	−0.0087 (12)
C10	0.0361 (15)	0.0474 (15)	0.0532 (17)	0.0056 (11)	0.0070 (12)	−0.0056 (12)
C11	0.0319 (13)	0.0514 (15)	0.0404 (15)	−0.0003 (11)	0.0095 (11)	−0.0023 (11)
C12	0.0378 (15)	0.0454 (15)	0.0516 (17)	−0.0024 (11)	0.0075 (12)	0.0027 (12)
C13	0.0344 (14)	0.0513 (16)	0.0498 (16)	0.0056 (12)	0.0057 (12)	0.0061 (12)
C14	0.072 (2)	0.0483 (18)	0.129 (3)	0.0055 (16)	0.009 (2)	−0.0209 (18)
C15	0.0331 (14)	0.0484 (15)	0.0519 (17)	0.0025 (11)	0.0061 (12)	−0.0009 (12)
C16	0.0342 (14)	0.0410 (13)	0.0400 (14)	−0.0017 (10)	0.0050 (11)	0.0034 (11)
C17	0.0350 (14)	0.0425 (14)	0.0450 (15)	−0.0034 (11)	0.0040 (11)	0.0025 (12)
C18	0.0394 (15)	0.0420 (14)	0.0420 (15)	−0.0020 (11)	0.0081 (11)	0.0037 (11)
C19	0.0559 (17)	0.0566 (17)	0.0601 (19)	0.0004 (13)	0.0140 (14)	−0.0072 (14)
C20	0.0500 (17)	0.0547 (17)	0.069 (2)	−0.0153 (13)	0.0060 (14)	−0.0055 (14)
C21	0.0302 (14)	0.0433 (14)	0.0566 (18)	−0.0069 (11)	0.0027 (12)	0.0102 (12)
C22	0.0435 (16)	0.0712 (19)	0.0592 (19)	−0.0060 (14)	0.0028 (14)	0.0157 (15)
C23	0.0453 (18)	0.085 (2)	0.082 (2)	−0.0057 (16)	−0.0090 (17)	0.0290 (19)
C24	0.0403 (18)	0.059 (2)	0.123 (3)	0.0019 (14)	0.009 (2)	0.023 (2)
C25	0.0491 (19)	0.0482 (17)	0.106 (3)	−0.0026 (13)	0.0195 (18)	0.0006 (16)
C26	0.0462 (17)	0.0502 (16)	0.066 (2)	−0.0015 (12)	0.0085 (14)	0.0014 (14)

### Geometric parameters (Å, °)

**Table:**

N1—O1	1.218 (3)	C8—C9	1.396 (3)
N1—O2	1.218 (3)	C9—C10	1.379 (3)
N1—C3	1.469 (3)	C10—C11	1.392 (3)
N2—O6	1.171 (4)	C10—H10	0.9300
N2—O5	1.238 (3)	C11—C12	1.385 (3)
N2—C13	1.473 (3)	C11—C15	1.460 (3)
N3—C15	1.270 (3)	C12—C13	1.376 (3)
N3—C16	1.399 (3)	C12—H12	0.9300
N4—C17	1.396 (3)	C14—H14A	0.9600
N4—N5	1.410 (3)	C14—H14B	0.9600
N4—C21	1.427 (3)	C14—H14C	0.9600
N5—C18	1.377 (3)	C15—H15	0.9300
N5—C20	1.471 (3)	C16—C18	1.356 (3)
O3—C8	1.370 (3)	C16—C17	1.447 (3)
O3—C7	1.423 (3)	C18—C19	1.487 (3)
O4—C9	1.365 (3)	C19—H19A	0.9600
O4—C14	1.435 (3)	C19—H19B	0.9600
O7—C17	1.237 (3)	C19—H19C	0.9600
C1—C2	1.380 (3)	C20—H20A	0.9600
C1—C6	1.386 (3)	C20—H20B	0.9600
C1—H1	0.9300	C20—H20C	0.9600
C2—C3	1.379 (3)	C21—C22	1.384 (4)
C2—H2	0.9300	C21—C26	1.384 (4)
C3—C4	1.371 (3)	C22—C23	1.382 (4)
C4—C5	1.374 (3)	C22—H22	0.9300
C4—H4	0.9300	C23—C24	1.369 (5)
C5—C6	1.385 (3)	C23—H23	0.9300
C5—H5	0.9300	C24—C25	1.376 (5)
C6—C7	1.504 (3)	C24—H24	0.9300
C7—H7A	0.9700	C25—C26	1.385 (4)
C7—H7B	0.9700	C25—H25	0.9300
C8—C13	1.393 (3)	C26—H26	0.9300
			
O1—N1—O2	122.8 (2)	C11—C12—H12	120.7
O1—N1—C3	118.3 (2)	C12—C13—C8	123.4 (2)
O2—N1—C3	118.9 (2)	C12—C13—N2	117.6 (2)
O6—N2—O5	122.4 (3)	C8—C13—N2	119.0 (2)
O6—N2—C13	120.1 (3)	O4—C14—H14A	109.5
O5—N2—C13	117.5 (3)	O4—C14—H14B	109.5
C15—N3—C16	118.5 (2)	H14A—C14—H14B	109.5
C17—N4—N5	109.92 (18)	O4—C14—H14C	109.5
C17—N4—C21	124.28 (19)	H14A—C14—H14C	109.5
N5—N4—C21	120.02 (19)	H14B—C14—H14C	109.5
C18—N5—N4	105.89 (18)	N3—C15—C11	123.1 (2)
C18—N5—C20	120.4 (2)	N3—C15—H15	118.5
N4—N5—C20	115.20 (19)	C11—C15—H15	118.5
C8—O3—C7	114.45 (18)	C18—C16—N3	124.0 (2)
C9—O4—C14	117.2 (2)	C18—C16—C17	108.3 (2)
C2—C1—C6	120.7 (2)	N3—C16—C17	127.7 (2)
C2—C1—H1	119.6	O7—C17—N4	123.5 (2)
C6—C1—H1	119.6	O7—C17—C16	131.9 (2)
C3—C2—C1	118.6 (2)	N4—C17—C16	104.6 (2)
C3—C2—H2	120.7	C16—C18—N5	110.6 (2)
C1—C2—H2	120.7	C16—C18—C19	128.2 (2)
C4—C3—C2	122.1 (2)	N5—C18—C19	121.3 (2)
C4—C3—N1	118.8 (2)	C18—C19—H19A	109.5
C2—C3—N1	119.1 (2)	C18—C19—H19B	109.5
C3—C4—C5	118.3 (2)	H19A—C19—H19B	109.5
C3—C4—H4	120.8	C18—C19—H19C	109.5
C5—C4—H4	120.8	H19A—C19—H19C	109.5
C4—C5—C6	121.5 (2)	H19B—C19—H19C	109.5
C4—C5—H5	119.2	N5—C20—H20A	109.5
C6—C5—H5	119.2	N5—C20—H20B	109.5
C5—C6—C1	118.6 (2)	H20A—C20—H20B	109.5
C5—C6—C7	118.2 (2)	N5—C20—H20C	109.5
C1—C6—C7	123.1 (2)	H20A—C20—H20C	109.5
O3—C7—C6	110.6 (2)	H20B—C20—H20C	109.5
O3—C7—H7A	109.5	C22—C21—C26	120.1 (2)
C6—C7—H7A	109.5	C22—C21—N4	121.2 (2)
O3—C7—H7B	109.5	C26—C21—N4	118.7 (2)
C6—C7—H7B	109.5	C23—C22—C21	119.3 (3)
H7A—C7—H7B	108.1	C23—C22—H22	120.3
O3—C8—C13	120.7 (2)	C21—C22—H22	120.3
O3—C8—C9	122.1 (2)	C24—C23—C22	120.9 (3)
C13—C8—C9	116.9 (2)	C24—C23—H23	119.6
O4—C9—C10	123.9 (2)	C22—C23—H23	119.6
O4—C9—C8	115.6 (2)	C23—C24—C25	119.9 (3)
C10—C9—C8	120.4 (2)	C23—C24—H24	120.0
C9—C10—C11	121.3 (2)	C25—C24—H24	120.0
C9—C10—H10	119.4	C24—C25—C26	120.1 (3)
C11—C10—H10	119.4	C24—C25—H25	120.0
C12—C11—C10	119.3 (2)	C26—C25—H25	120.0
C12—C11—C15	122.7 (2)	C21—C26—C25	119.8 (3)
C10—C11—C15	118.0 (2)	C21—C26—H26	120.1
C13—C12—C11	118.7 (2)	C25—C26—H26	120.1
C13—C12—H12	120.7		
			
C17—N4—N5—C18	−9.2 (2)	O3—C8—C13—N2	−8.4 (4)
C21—N4—N5—C18	−163.58 (19)	C9—C8—C13—N2	177.2 (2)
C17—N4—N5—C20	−145.0 (2)	O6—N2—C13—C12	135.8 (4)
C21—N4—N5—C20	60.7 (3)	O5—N2—C13—C12	−43.2 (4)
C6—C1—C2—C3	0.6 (4)	O6—N2—C13—C8	−42.7 (5)
C1—C2—C3—C4	0.8 (4)	O5—N2—C13—C8	138.2 (3)
C1—C2—C3—N1	−178.0 (2)	C16—N3—C15—C11	−176.8 (2)
O1—N1—C3—C4	−172.0 (2)	C12—C11—C15—N3	11.5 (4)
O2—N1—C3—C4	5.4 (3)	C10—C11—C15—N3	−168.0 (2)
O1—N1—C3—C2	6.8 (3)	C15—N3—C16—C18	−161.3 (2)
O2—N1—C3—C2	−175.8 (2)	C15—N3—C16—C17	21.5 (4)
C2—C3—C4—C5	−1.3 (4)	N5—N4—C17—O7	−173.0 (2)
N1—C3—C4—C5	177.5 (2)	C21—N4—C17—O7	−20.0 (4)
C3—C4—C5—C6	0.3 (4)	N5—N4—C17—C16	6.8 (2)
C4—C5—C6—C1	1.0 (4)	C21—N4—C17—C16	159.8 (2)
C4—C5—C6—C7	−176.9 (3)	C18—C16—C17—O7	178.0 (3)
C2—C1—C6—C5	−1.5 (4)	N3—C16—C17—O7	−4.5 (4)
C2—C1—C6—C7	176.4 (3)	C18—C16—C17—N4	−1.8 (3)
C8—O3—C7—C6	−178.1 (2)	N3—C16—C17—N4	175.7 (2)
C5—C6—C7—O3	−164.2 (2)	N3—C16—C18—N5	178.4 (2)
C1—C6—C7—O3	17.9 (4)	C17—C16—C18—N5	−4.0 (3)
C7—O3—C8—C13	116.4 (3)	N3—C16—C18—C19	−1.7 (4)
C7—O3—C8—C9	−69.5 (3)	C17—C16—C18—C19	175.9 (2)
C14—O4—C9—C10	−11.0 (4)	N4—N5—C18—C16	8.1 (3)
C14—O4—C9—C8	171.8 (3)	C20—N5—C18—C16	141.0 (2)
O3—C8—C9—O4	2.0 (3)	N4—N5—C18—C19	−171.8 (2)
C13—C8—C9—O4	176.2 (2)	C20—N5—C18—C19	−39.0 (3)
O3—C8—C9—C10	−175.3 (2)	C17—N4—C21—C22	−123.6 (3)
C13—C8—C9—C10	−1.1 (4)	N5—N4—C21—C22	26.8 (3)
O4—C9—C10—C11	−174.8 (2)	C17—N4—C21—C26	55.4 (3)
C8—C9—C10—C11	2.3 (4)	N5—N4—C21—C26	−154.1 (2)
C9—C10—C11—C12	−1.2 (4)	C26—C21—C22—C23	−0.6 (4)
C9—C10—C11—C15	178.3 (2)	N4—C21—C22—C23	178.4 (2)
C10—C11—C12—C13	−1.1 (4)	C21—C22—C23—C24	−0.2 (4)
C15—C11—C12—C13	179.4 (2)	C22—C23—C24—C25	0.6 (4)
C11—C12—C13—C8	2.3 (4)	C23—C24—C25—C26	−0.1 (4)
C11—C12—C13—N2	−176.2 (2)	C22—C21—C26—C25	1.1 (4)
O3—C8—C13—C12	173.1 (2)	N4—C21—C26—C25	−178.0 (2)
C9—C8—C13—C12	−1.2 (4)	C24—C25—C26—C21	−0.7 (4)

### Hydrogen-bond geometry (Å, °)

**Table:**

*D*—H···*A*	*D*—H	H···*A*	*D*···*A*	*D*—H···*A*
C15—H15···O7	0.93	2.35	2.997 (3)	127
C4—H4···O7^i^	0.93	2.56	3.447 (3)	159

Symmetry codes: (i) *x*−1/2, −*y*−1/2, *z*−1/2.
